# Eligibility for amyloid targeting therapies among primary care patients with cognitive symptoms

**DOI:** 10.1186/s13195-026-02019-2

**Published:** 2026-03-21

**Authors:** Beata Borgström Bolmsjö, Josef Barbosa Djärf, Danielle van Westen, Suzanne E. Schindler, Ayesha Fawad, Lyduine Collij, Ruben Smith, Niklas Mattsson-Carlgren, Erik Stomrud, Pontus Tideman, Oskar Hansson, Sebastian Palmqvist

**Affiliations:** 1 Center for Primary Health Care Research, Department of Clinical Sciences in Malmo, Lund University, Malmo, Sweden; 2 Memory Clinic, Skane University Hospital, Malmo, Sweden; 3 Clinical Memory Research Unit, Department of Clinical Sciences, Lund University, Malmo, Sweden; 4 University Clinic Primary Care Skane, Region Skane, Sweden; 5 Diagnostic Radiology, Institution for Clinical Sciences, Lund University, Lund, Sweden; 6 Image and Function, Skane University Hospital, Lund, Sweden; 7 Department of Neurology, Washington University in St. Louis, St. Louis, Missouri USA; 8 Department of Radiology, Vrije Universiteit Amsterdam, Amsterdam UMC location VUmc, Amsterdam, Netherlands; 9 Brain Imaging, Amsterdam Neuroscience, Amsterdam, Netherlands

**Keywords:** Alzheimer’s disease, Primary care, Eligibility, Anti-amyloid therapy

## Abstract

**Background:**

Alzheimer’s disease (AD) is the most common cause of dementia and a growing healthcare challenge. Amyloid-targeting therapies (ATT) may slow progression, but implementation is limited by logistical and economic barriers. As primary care is the first contact for most patients with cognitive concerns, quantifying treatment eligibility in this setting is essential. The purpose of this study was to estimate the proportion of primary care patients presenting with cognitive symptoms who are eligible for ATT.

**Methods:**

This cohort study included patients presenting with cognitive symptoms in primary care across the region Skåne, in southern Sweden, recruited between January 2020 and April 2025. Stepwise exclusion criteria based on clinical diagnosis, comorbidities, and treatment contraindications were applied, in alignment with appropriate use recommendations for lecanemab and donanemab, respectively. Eligibility was further refined using CSF biomarkers (Aβ42/40 ratio), cognitive performance, and MRI findings.

**Results:**

In a full diagnostic work-up of 607 patients with sequential exclusions, 86 patients (14.2%) and 78 patients (12.8%) ultimately met the eligibility criteria for lecanemab and donanemab, respectively. Due to comorbidities, medication use, and age/BMI, around 1/3 of the original population was excluded. Most ineligible patients met more than one exclusion criterion. The eligible population was 63% female, mean age 77 years. Around 65% of the individuals had mild cognitive impairment (MCI), and 35% mild dementia.

**Conclusions:**

About 13-14% of primary care patients evaluated for cognitive complaints were eligible for ATT. Compared with clinical trials, the eligible population was older and consisted of more women.

**Trial registration:**

BioFINDERPrimary Care study (NCT06120361, Registration date 2 November 2023 https//biofinder.se).

**Supplementary Information:**

The online version contains supplementary material available at 10.1186/s13195-026-02019-2.

## Background

The amyloid-targeting therapies (ATT) lecanemab and donanemab received U.S. Food and Drug Administration (FDA) approval in 2023 and 2024 [1, 2] and have since been authorized in several major markets, including Japan and China [3–6]. The European Medicines Agency (EMA) approved lecanemab in November 2024 [7] for mild cognitive impairment or early AD, followed by donanemab in July 2025 [8]. Affordability concerns have emerged as projections suggest that widespread eligibility for ATTs could impose substantial budgetary pressures [9, 10]. As a result, several national agencies recommend caution until more comprehensive health-economic assessments are available [11].

The referral pattern and patient population at specialized memory clinics are likely to change gradually as ATT becomes more widely adopted. For example, many secondary and tertiary clinics have historically only accepted patients with uncommon dementias or atypical AD, rather than the large group of patients who may be suitable candidates for ATT. To accurately estimate overall ATT eligibility, assessment must begin with individuals in primary care, as they represent the potential candidates for referral for further evaluation and potential treatment. The inclusion criteria in clinical trials are typically highly restrictive, often including only patients with few comorbidities or medications that may interact with the specific treatment [12–14]. In contrast, primary care populations frequently present with multimorbidity and polypharmacy, factors that substantially reduce eligibility for ATT [15]. It is essential to estimate what proportion of primary care patients would meet eligibility criteria, as this is the key to understanding the broader impact of ATT on diagnostic capacity, staffing needs, and healthcare costs.

The aim of this study was to characterize and to determine the proportion of patients presenting to primary care with cognitive symptoms who would be eligible for ATTs using the full diagnostic work-up of today.

## Methods

This observational study was conducted in accordance with the regulatory requirements and the ethical principles of the Declaration of Helsinki as adopted by the 18th World Medical Assembly. The study was approved by the Swedish Ethical Review Authority. An informed consent was signed and collected before enrolment in the study.

The study report adheres to the STROBE (Strengthening the Reporting of Observational Studies in Epidemiology) recommendations (Table S1) [16].

### Setting and participants

The primary care pathway for patients with cognitive symptoms in Sweden is described in detail in the Supplementary Material. Participants were recruited consecutively at 23 primary care centers in southern Sweden between January 15, 2020 and April 30, 2025, as part of the prospective BioFINDER-Primary Care study (NCT06120361; Registration date: 2 November 2023, https://biofinder.se) [17–19]. Primary care physicians included patients presenting with cognitive complaints according to the following inclusion criteria: (1) the patient seeks medical care in primary care because of cognitive symptoms experienced by the patient and/or next of kin, or the primary care physician suspects a progressive neurodegenerative disorder; (2) age ≥ 40 years; and (3) cognitive impairment classified as subjective cognitive decline (SCD), mild cognitive impairment (MCI), or dementia. Exclusion criteria were: (1) previously established diagnosis of dementia; (2) significant unstable systemic illness precluding study participation; (3) current significant alcohol or substance misuse; (4) unwillingness to undergo further examinations; (5) acute-onset cognitive impairment due to stroke; and (6) cognitive impairment that, with high certainty as assessed by the primary care physician, could be explained by another condition (e.g., psychotic disorder, depression, or alcohol misuse). Beyond the above-mentioned inclusion and exclusion criteria, the cohort for the present study comprised participants from the BioFINDER-Primary Care study who either (1) underwent MRI at baseline, or (2) had contraindications to MRI. A flow chart of participant inclusion is provided in Figure S1. Primary care physicians were encouraged to include patients presenting with cognitive complaints, subject to the predefined inclusion and exclusion criteria.

### Variables and data sources

The proportion of individuals eligible for treatment with lecanemab or donanemab was assessed by identifying patients who met the Appropriate Use Recommendation (AUR) inclusion criteria for treatment described by Cummings et al. for lecanemab [20] and Rabinovici et al. for donanemab [21] (Table 1).

**Table 1:** Eligibility criteria for amyloid targeting therapies

Appropriate use recommendations (AUR) lecanemab	Appropriate use recommendations (AUR) donanemab	Criteria operationalized in the BioFINDER-PC study, and in sensitivity analyses
*Inclusion criteria*		
Clinical diagnosis of MCI or mild AD dementia (In Clarity-AD MMSE 22–30)	Same as AUR lecanemab (In TRAILBLAZER-ALZ-phase 2 trial MMSE 20–28)	MCI and mild dementia diagnoses were determined using cognitive test results, biomarkers, and clinical assessments, reviewed in weekly consensus meetings with Memory Clinic specialists and neuropsychologists
Positive CSF or PET indicative of AD	Same as AUR lecanemab	Either with cerebrospinal fluid (CSF) Lumipulse Aβ42/40 ratio with a cutoff of ≤ 0.072 indicating a positive result or visual read of [18 F]flutemetamol PET.
Physician judgement was used for patients outside the 50–90 year age range	Physician judgement was used for patients outside the 60–85 year age range	Participants in the BioFINDER-Primary Care study were of age ≥ 40 years. Analyses were made for AUR lecanemab 50–90 years and AUR donanemab 60–85 years, and all ages respectively
Body mass index (BMI) > 17 and < 35 or physician judgement outside range	No BMI limit	Participants in the BioFINDER-Primary Care study had no BMI limit. Analyses were made for AUR lecanemab with and without BMI limits, and for donanemab with no BMI limits
May be on acetylcholinesterase inhibitor (donepezil, rivastigmine, galantamine) or memantine or both. Patients may not currently be on aducanumab or lecanemab;	Same as AUR lecanemab	Yes
May be on standard care for other medical illnesses (see below for exclusions)	Same as AUR lecanemab	Same as AUR lecanemab and AUR donanemab
Have a care partner that can support treatment and understands benefits and harms of the treatment*	Same as AUR lecanemab Treated patients should understand that the effect of donanemab on the ability to have children or its effect on the unborn fetus are unknown In the opinion of the clinician, have adequate literacy, vision, and hearing for cognitive testing*	Yes. All included patients had support for participating
Clinical apolipoprotein E genotyping is performed prior to initiating treatment to assess an individual’s risk of ARIA	Same as AUR lecanemab	Same as AUR lecanemab and AUR donanemab
*Exclusion criteria*		
Any medical, neurologic, or psychiatric condition that may be contributing to the cognitive impairment or any non-AD MCI or dementia	Same as AUR lecanemab and patients with autosomal dominant AD, if the mutation is associated with high prevalence and burden of cerebral amyloid angiopathy (CAA). Patients with AD due to Down syndrome	Same as AUR lecanemab
MRI contraindications	Same as AUR lecanemab	Same as AUR lecanemab and AUR donanemab
More than 4 microhemorrhages (≤ 10 mm); a single macrohemorrhage (> 10 mm), an area of superficial siderosis; evidence of vasogenic edema; >2 lacunar infarcts or stroke involving a major vascular territory; Fazekas score of 3, evidence of amyloid beta-related angiitis, cerebral amyloid angiopathy-related inflammation, or other major intracranial pathology that may cause cognitive impairment	Evidence of amyloid-related imaging abnormalities of edema; more than four microhemorrhages; any area of superficial siderosis; a single macrohemorrhage > 10 mm; severe white matter disease; territorial infarcts > 1 cm; or more than two lacunar infarcts. Evidence of amyloid beta–related angiitis, cerebral amyloid angiopathy–related inflammation, cerebral contusion, encephalomalacia, brain aneurysms or other vascular malformations, central nervous system infection, or brain tumors (except for small meningiomas or arachnoid cysts)	Fazekas grade 3, > 2 lacunar infarctions, > 4 microbleeds, any hemorrhage > 10 mm and cortical siderosis were excluded. In addition, participants with infarctions > 2 cm in any dimension, were excluded based on clinical consensus Sensitivity analysis where no microbleed was excluded was made
Recent history (within 12 months) of stroke or transient ischemic attacks	Same as AUR lecanemab	Same as AUR lecanemab and AUR donanemab
Any history of seizures	Same as AUR lecanemab	Active prescription of anti-epileptic medication
Mental illness (e.g., psychosis, severe depression) that interferes with comprehension of the requirements, potential benefit, and potential harms of treatment and are considered by the physician to render the patient unable to comply with management requirements	Same as AUR lecanemab	Same as AUR lecanemab and AUR donanemab
Any history of immunologic disease (e.g., lupus erythematosus, rheumatoid arthritis, Crohn’s disease) or systemic treatment with immunosuppressants, immunoglobulins, or monoclonal antibodies.	Same as AUR lecanemab	Same as AUR lecanemab and AUR donanemab Sensitivity analyses were made with a more inclusive approach.
Patients with a bleeding disorder that is not under adequate control (including a platelet count < 50,000 or international normalized ratio [INR] > 1.5 for participants who are not on anticoagulant)	Same as AUR lecanemab	Amyloid PET was used when CSF sampling was contraindicated due to bleeding disorders or platelet counts < 50,000: Patients undergoing amyloid PET for other reasons were confirmed to have no bleeding disorder or platelet counts < 50,000.
Patients on anticoagulants (warfarin, dabigatran, edoxaban, rivaroxaban, apixaban, betrixaban, or heparin) should not receive lecanemab; tPA should not be administered to individuals on lecanemab	Same as AUR lecanemab	Same as AUR lecanemab and AUR donanemab
Unstable medical conditions that may affect or be affected by lecanemab therapy	Same as AUR lecanemab Active cancer that interferes with the ability to comply with donanemab treatment**	Same as AUR lecanemab and AUR donanemab. Sensitivity analyses were made with a more inclusive approach.

* It is not possible to evaluate this aspect within the BioFINDER study, as the physician’s decision regarding inclusion is based exclusively on the participant’s ability to undergo the required study assessments. Given that the ATT medication is not yet available in Sweden, this parameter cannot be assessed for the patients enrolled in the present study

**Active cancer defined as history of cancer 5 years, except for non-metastatic basal and/or squamous cell carcinoma of the skin, nonprogressive prostate cancer, or other cancers with low risk of recurrence or spread

The study procedures are briefly described below. A detailed description of the methodology has been published previously in the study protocol [19]. The first visit was conducted at the patients’ local primary care unit, where all patient information was collected by a health care professional. Underlying health conditions and medications were registered, and height and weight were measured. The participants completed cognitive testing, filled out questionnaires about their social and personal background, activity of daily living (ADL) and cognitive symptoms. All patients were then referred to the Memory Clinic at Skåne University Hospital, Malmö, Sweden, for further cognitive testing, MRI, lumbar puncture, and evaluation by a dementia expert.

Cognitive status was determined at weekly consensus meetings involving neuropsychologists and dementia specialists (including the treating physician), with a detailed description of the procedure provided in the Supplementary Material.

Amyloid status was primarily determined using CSF Aβ42/40 (≤ 0.072 for positivity; FDA-cleared cut-off) measured with the Lumipulse assay. In participants with contraindications to lumbar puncture or who declined the procedure, 18 F-flutemetamol PET was performed instead [22]. The most common contraindication was ongoing anticoagulant therapy for atrial fibrillation, where patients with prior stroke or CHA₂DS₂-VASc score > 4 underwent PET instead. PET scans were visually rated by two independent experienced readers blinded to clinical data, and discordant reads resolved by consensus. Amyloid status was treated as a binary composite reference standard (CSF or PET), with CSF- and PET-based classifications considered analytically equivalent. Participants underwent either CSF or PET, but not both. AD etiology was determined according to the clinical International Working Group (IWG) criteria for probable or established AD [23], with detailed description in the Supplementary material.

Age and BMI criteria were applied using strict AUR cut-offs in the primary analysis of eligibility (Table 1). Contraindications related to medications and comorbidities were operationalized based on the AUR for lecanemab and donanemab, using diagnostic and prescription register data from the medical records according to the World Health Organization’s International Classification of Diseases (ICD) [24] and the Anatomical Therapeutic Chemical (ATC) classification system [25]. Full ICD-10 and ATC code lists together with decision rules used for exclusion are provided in Table S2. Due to the lack of temporal specificity and limited operationalizability of the AUR criterion “any history of seizures” in medical records, seizure-related contraindications were operationalized as active prescriptions of antiepileptic medication. Autoimmune contraindications were operationalized according to AUR criteria as any recorded systemic autoimmune or inflammatory disease and/or ongoing systemic immunosuppressive therapy. Cancer related contraindications were operationalized using a conservative 5-year exclusion window, excluding non-melanoma skin cancer and carcinoma in situ. Anticoagulant treatment was identified from medical records as an active prescription of any anticoagulant classified under ATC code B01A. Patients with platelet counts < 50 × 10^9^/L were considered ineligible for antibody treatment.

These medical exclusion criteria were evaluated by a specialist (BBB), and any unclear cases were discussed with a second specialist (SP). Pragmatic interpretations of the AUR were further discussed with a senior associate consultant (SES) with direct clinical experience in ATT implementation.

### MRI, imaging and assessment

Contraindications for MRI included pacemaker, severe claustrophobia, implanted magnetic metal objects, metallic fragments in the eye, or metal shrapnel near critical structures such as major blood vessels and nerves. MRI was performed on a 3T Siemens MAGNETOM Vida or a 3T Siemens MAGNETOM Prisma. The MR protocol included a 3D T1 weighted sequence, 3D fluid attenuated inversion recovery (FLAIR), and susceptibility weighted imaging (SWI) or quantitative susceptibility weighted mapping (QSM); in the latter case, images with the strongest susceptibility weighting were used to assess the number and location of microbleeds and the presence of cortical siderosis. Visual assessment was performed by a senior neuroradiologist (DvW). Assessment of white matter hyperintensities (WMH) was performed using the Fazekas scale as modified by Prins et al. [26], of lacunes based on the STRIVE criteria, original and updated [27, 28] and of microbleeds based on the MARS rating scale [29]. Infarctions were visually identified and measured including the surrounding gliosis, whereas gliosis was not included in the assessment of WMH. 

Participants with Fazekas grade 3, > 2 lacunes, > 4 microbleeds, any hemorrhage > 10 mm and cortical siderosis were excluded as recommended by Cummings et al. [20]. In addition, participants with infarctions > 2 cm in any dimension, were excluded based on clinical consensus. Of note, some participants met several of these exclusion criteria. Other MRI abnormalities assessed according to AUR were vasogenic edema, cerebral amyloid angiopathy-related inflammation (CAA-ri) or Aβ-related angiitis (ABRA), and cortical siderosis.

### Sensitivity analyses

Several sensitivity analyses were conducted, each modifying a single interpretation of the AUR criteria. while all other eligibility criteria were kept constant (Table S2 and S4). In the first analyses (sensitivity analyses 1–3), a maximally permissive physician judgment scenario was examined by removing age and BMI restrictions accordingly. In the fourth analysis, autoimmune-related exclusions were redefined using a more permissive clinical definition, restricting exclusion to active autoimmune disease requiring systemic immunosuppressive treatment. In a fifth sensitivity analysis, malignancy-related exclusions were limited to clinically significant unstable disease, consistent with the BioFINDER-Primary Care exclusion criterion. Under this definition, participants with significant active cancer were considered already excluded at cohort entry, and no additional exclusions for malignancy were applied. A sixth sensitivity analysis was made with no exclusion for anticoagulation treatment. The seventh and last additional sensitivity analysis was made to assess the potential impact of the higher sensitivity of 3T susceptibility-weighted imaging used in this study for detecting cerebral microbleeds, where the exclusion criteria based on microbleeds was removed.

### Statistical methods

Descriptive analyses were performed using Excel, IBM SPSS Statistics, and R version 4.3. Group differences for continuous variables were assessed using independent-samples t-tests, while differences in categorical variables were analyzed using Pearson’s chi-square (χ²) tests. All statistical comparisons were two-tailed with a significant p-value set at *p* < 0.05.

Intra-rater reliability of visual MRI ratings was evaluated in 83 randomly selected participants re-assessed after six months by the same blinded neuroradiologist (DvW). Weighted Cohen’s kappa with quadratic weights was used for Fazekas scores, and unweighted Cohen’s kappa for all other dichotomous MRI variables (*p* < 0.05). Intra-rater agreement was excellent across all MRI exclusion criteria (κ = 0.85–1.00; Table S6). Inter-rater agreement for amyloid PET was assessed using Cohen’s kappa (*p* < 0.05), where agreement was shown excellent (κ = 0.91; 95% CI 0.85–0.98).

## Results

### Participants

Our cohort consisted of 607 individuals with a mean (SD) age of 75.9 (7.5) years (Table 2).

**Table 2:** Descriptive statistics of population and exclusion criteria based on amyloid status

	All	Amyloid positive	Amyloid negative
Total population, n (%)	607 (100%)	320 (52.7%)	287 (47.3%)
Demographics			
Age, years, mean ± SD	75.9 ± 7.5	77.5 ± 6.8	74.2 ± 7.9*
Age < 50 or > 90 years, n (%)	10 (1.6%)	4 (1.2%)	6 (2.1%)
Age < 60 or > 85 years, n (%)	72 (11.9%)	47 (14.7%)	25 (8.7%)*
Sex, female, n (%)	320 (52.7%)	190 (59.4%)	130 (45.3%)*
BMI, mean ± SD	25.9 ± 4.2	24.9 ± 3.8	26.9 ± 4.3*
BMI < 17 or > 35, n (%)	20 (3.3%)	5 (1.6%)	15 (5.2%)*
Education level, years, mean ± SD	11.4 ± 3.2	11.2 ± 3.2	11.5 ± 3.3
Cognitive assessments			
MMSE, mean ± SD	26.4 ± 3.2	25.9 ± 3.3	27.0 ± 2.8*
MoCA, mean ± SD	21.9 ± 3.9	21.3 ± 3.9	22.5 ± 3.9*
CDR-SOB, mean ± SD	1.5 ± 1.9	1.7 ± 1.9	1.2 ± 1.9*
FAQ, mean ± SD	6.8 ± 6.2	7.6 ± 6.3	5.9 ± 6.0*
Cognitive stage			
Subjective cognitive decline (SCD), n (%)	207 (34.1%)	73 (22.8%)	134 (46.7%)*
Mild cognitive impairment (MCI), n (%)	261 (43.0%)	147 (45.9%)	114 (39.7%)
Mild dementia, n (%)	124 (20.4%)	89 (27.8%)	35 (12.2%)*
Moderate dementia, n (%)	15 (2.5%)	11 (3.4%)	4 (1.4%)
Clinical Consensus Diagnosis			
No clinical diagnosis or SCD, n (%)	199 (32.8%)	71 (22.2%)	128 (44.6%)*
Alzheimer’s disease, n (%)	204 (33.6%)	204 (63.8%)	0 (0%)*
Vascular cognitive impairment, n (%)	67 (11.0%)	10 (3.1%)	57 (19.9%)*
Unspecified MCI or dementia, n (%)	100 (16.5%)	21 (6.6%)	79 (27.5%)*
Other neurodegenerative disease, n (%)	37 (6.1%)	14 (4.4%)	23 (8.0%)
Medications and comorbidities			
Anticoagulant treatment, n (%)	98 (16.1%)	49 (15.3%)	49 (17.1%)
Autoimmune disorder, n (%)	54 (8.9%)	26 (8.1%)	28 (9.8%)
Active cancer^1^, n (%)	24 (4.0%)	12 (3.8%)	12 (4.2%)
Stroke/TIA within 12 m, n (%)	23 (3.8%)	12 (3.8%)	11 (3.8%)
Antiepileptic medications, n (%)	13 (2.1%)	7 (2.2%)	6 (2.1%)
Any exclusion criterion regarding medication or comorbidity, n (%)	180 (29.7%)	89 (27.8%)	91 (31.7%)
Neuroimaging			
MRI contraindication, n (%)	35 (5.8%)	15 (4.7%)	20 (7.0%)
Fazekas 3, n (%)	156 (25.7%)	92 (28.8%)	64 (22.3%)
> 2 Lacunes n (%)	37 (6.1%)	17 (5.3%)	20 (7.0%)
> 4 Microbleeds, n (%)	54 (8.9%)	34 (10.6%)	20 (7.0%)
Macrohemorrhage > 10 mm, n (%)	14 (2.3%)	7 (2.2%)	7 (2.4%)
Cortical siderosis, n (%)	16 (2.6%)	12 (3.8%)	4 (1.4%)
Vascular edema, n (%)	1 (0.2%)	1 (0.3%)	0 (0%)
Other major neuropathology^2^, n (%)	29 (4.8%)	15 (4.7%)	14 (4.9%)
Any exclusion criterion regarding pathological neuroimaging, n (%)	196 (32.3%)	117 (36.6%)	79 (27.5%)*
Genetics			
* APOE* ε4 non-carrier, n (%)	362 (59.6%)	132 (41.3%)	230 (80.1%)*
* APOE* ε4 homozygous, n (%)	34 (5.6%)	33 (10.3%)	1 (0.3%)*
* APOE* ε4 heterozygous, n (%)	211 (34.8%)	155 (48.4%)	56 (19.5%)*
Eligibility			
Eligible for lecanemab ATT, n (%)^3^	75 (12.4%)	75 (23.4%)	0 (0%)
Eligible for donanemab ATT, n (%)^3^	68 (11.2%)	68 (21.3%)	0 (0%)

*BMI* Body Mass Index, *MMSE* Mini Mental State Examination, *MoCA* Montreal Cognitive Assessment, *CDR-SOB* Clinical Dementia Rating -Sum of Boxes, *FAQ* Functional Activities Questionnaire, *APOE* Apolipoprotein-E, *ATT* Amyloid Targeting Therapy

*p-value < 0.05 for comparisons between amyloid-positive and amyloid-negative groups: χ²-test for categorical variables, and independent-samples t-test for continuous variables

^1^Active cancer defined as history of cancer 5 years, except for non-metastatic basal and/or squamous cell carcinoma of the skin, nonprogressive prostate cancer, or other cancers with low risk of recurrence or spread

^2^ Including large infarctions >20 mm

^3^ The eligible population excluding* APOE *ε4 homozygous participants

In total, 845 individuals were recruited, of whom 238 were excluded from the present analysis (e.g. due to missing MRI sequences, refusal, contraindications, incomplete biomarker ascertainment), as presented in Figure S1. Baseline characteristics (age, sex, BMI and MMSE) were available for approximately 160 of the excluded individuals. As shown in Table S3, no major differences were observed between included and excluded participants with respect to these variables.

Amyloid status was available for all 607 participants: 523 based on CSF (283 positive) and 84 based on amyloid PET (37 positive). The proportion of amyloid-positives was 53% (*n* = 320) and this group was significantly older, included a higher proportion of women, showed more cognitive impairment, and had a higher prevalence of *APOE ε4* homozygosity than the amyloid-negative group (all comparisons *p* < 0.05) (Table 2). Among amyloid positive individuals, around 20% fulfilled all additional eligibility criteria for ATT. Among those that were amyloid positive, the majority were also tau (T1) positive across different age groups (Table S7).

### ATT eligibility for lecanemab

**Fig. 1:**
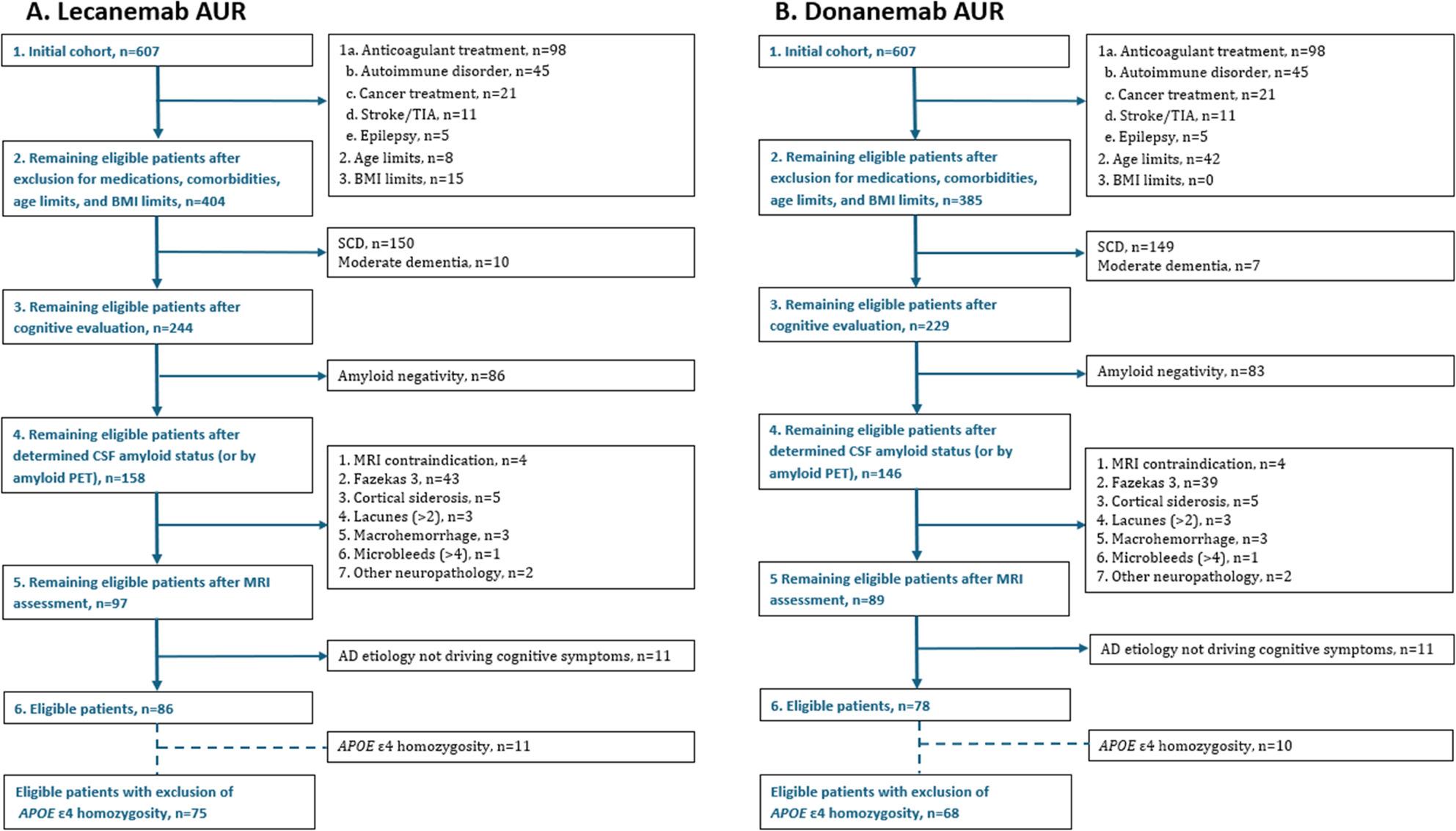
Eligibility flow chart for (**a**)lecanemab (**b**)donanemab. Stepwise representation of exclusions in the full diagnostic workup showing number of individuals eligible for lecanemab (**a**)and donanemab (**b**), according to their respective appropriate use recommendations. Each step corresponds to a specific exclusion criterion or assessment (see Table 1), with numbering, including lowercase letters, indicating hierarchical order based on the prevalence of each condition. Details within each step specify the particular criteria applied and the number of participants excluded

After excluding patients in the following order: (1) comorbidities and medications that could interfere with treatment (*n* = 180), (2) outside the appropriate age range (*n* = 8), (3) too high/low BMI (*n* = 15), 404 participants remained (Fig. 1A). Subsequently, patients with more severe cognitive status than mild dementia or with SCD were excluded (*n* = 160), leaving 244 individuals. Following, amyloid-negative participants were excluded (*n* = 86), with 158 amyloid-positive participants remaining. After evaluation of exclusions based on neuroradiology (*n* = 61) and specialist confirmation of AD etiology as underlying factor driving the cognitive symptoms (excluding *n* = 11), 86 patients remained eligible corresponding to 14.2% of the initial population. If also excluding *APOE ε4* homozygosity, following EMA’s guidelines [7], 75 (12.4%) patients were considered eligible for ATT (Fig. 1A).

### Differences when applying the appropriate use recommendations of lecanemab vs. donanemab

When applying donanemab AUR, which differs to lecanemab AUR with regards to BMI- and age limits (Table 1), the number of patients eligible for donanemab was 78, corresponding to 12.8% of the study population (Fig. 1B). After also excluding patients with *APOE ε4* homozygosity the number of eligible patients was 68 (11.2%).

### Characteristics of the eligible population

In the lecanemab eligible population, 63% were female, the mean (SD) age was 77 (6.4) years (Table 3). The mean (SD) MMSE score was 25 (3.4). Approximately two thirds were classified as having MCI, and one third as mild dementia. Among comorbidities, hypertension was present in almost half of the patients, and about 16% had diabetes. Characteristics of the donanemab eligible population were similar (Table 3).

**Table 3:** Descriptive statistics for ATT eligible populations

	Lecanemab	Donanemab
Total eligible population* n (%)	75 (100%)	68 (100%)
Demographics		
Age, years (mean ± SD)	77.0 ± 6.4	76.3 ± 5.1
Sex, female n (%)	47 (62.7%)	43 (63.2%)
BMI (mean ± SD)	24.0 ± 2.9	24.3 ± 3.9
Education level, years (mean ± SD)	11.2 ± 3.0	11.3 ± 3.1
Cognitive assessments		
MMSE (mean ± SD)	25.1 ± 3.4	25.0 ± 3.6
MoCA (mean ± SD)	20.5 ± 4.5	20.8 ± 4.2
CDR-SOB (mean ± SD)	2.0 ± 1.6	1.9 ± 1.6
FAQ (mean ± SD)	8.6 ± 6.3	8.2 ± 6.0
Cognitive stage		
Mild cognitive impairment (MCI), n (%)	47 (62.7%)	45 (66.2%)
Mild dementia n (%)	28 (37.3%)	23 (33.8%)
Neuroimaging		
Fazekas 0 (n, %)	0 (0.0%)	0 (0.0%)
Fazekas 1 (n, %)	55 (73.3%)	51 (75.0%)
Fazekas 2 (n, %)	20 (26.7%)	17 (25.0%)
1 Lacune (n, %)	7 (9.3%)	6 (8.8%)
2 lacunes (n, %)	2 (2.7%)	2 (2.9%)
1–2 Microbleeds (n, %)	6 (8.0)%	6 (8.8%)
3–4 Microbleeds (n, %)	6 (8.0%)	6 (8.8%)
Medications and comorbidities		
Diabetes (n, %)	12 (16.0%)	10 (14.7%)
Hypertension (n, %)	33 (44.0%)	32 (47.1%)
Hyperlipidemia (n, %)	18 (24.0%)	18 (26.5%)
Ischemic heart disease (n, %)	9 (12.0%)	9 (13.2%)
Genetics		
* APOE* ε4 non-carrier (n, %)	32 (42.7%)	28 (41.2%)
* APOE* ε4 heterozygous (n, %)	43 (57.3%)	40 (58.8%)

*BMI* Body Mass Index, *MMSE* Mini Mental State Examination, *MoCA* Montreal Cognitive Assessment, *CDR-SOB* Clinical Dementia Rating -Sum of Boxes, *FAQ* Functional Activities Questionnaire, *APOE* Apolipoprotein-E

*The eligible population excluding APOE ε4 homozygous participants

### Specific exclusion criteria

**Fig. 2:**
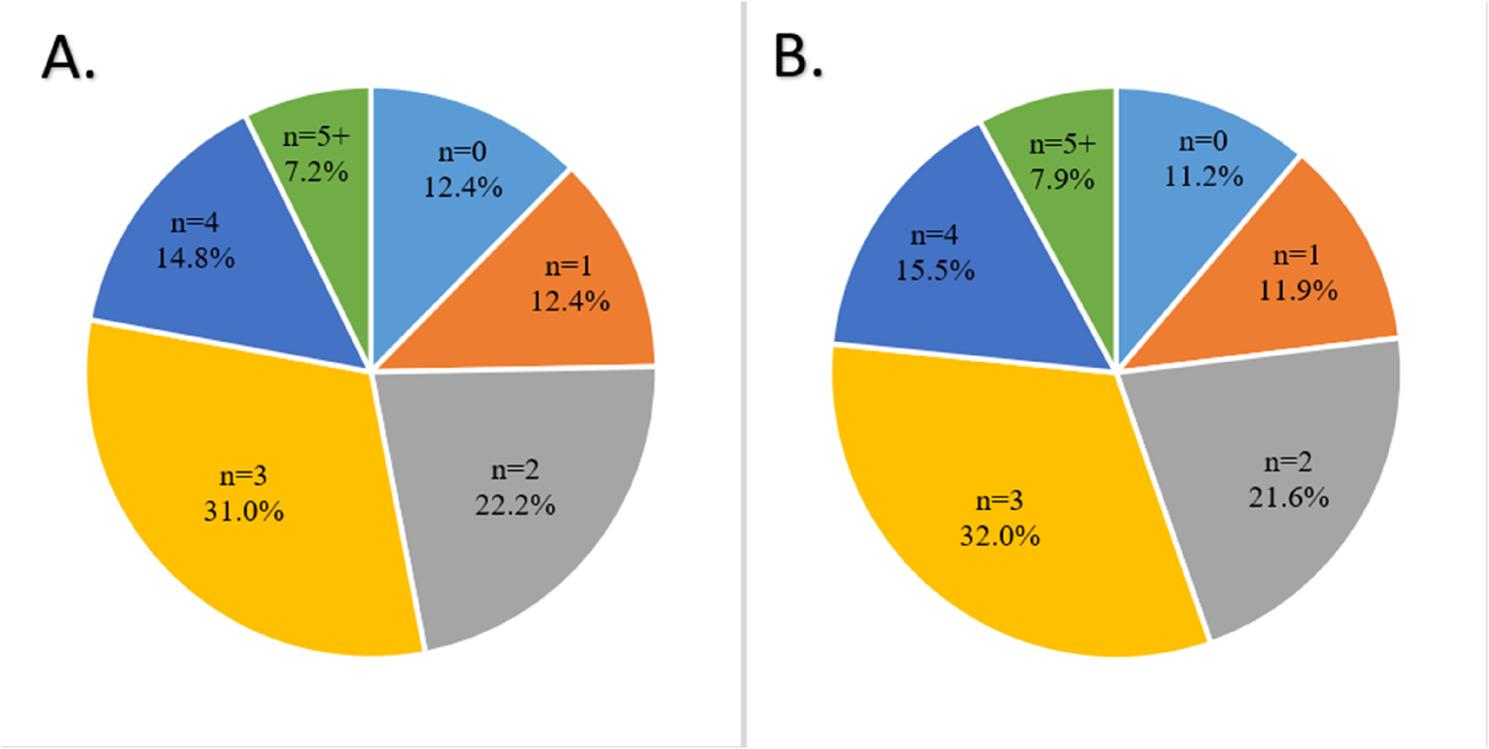
Distribution of the number of exclusion criteria per participant (n) across the study population for eligibility for (**a**) lecanemab and **b** donanemab

Table 2 summarizes the number of patients fulfilling each exclusion criterion and major exclusion groups. In total, 180 participants had any comorbidity or ongoing treatments incompatible with ATT, of whom 98 were receiving anticoagulant therapy. Most participants met multiple exclusion criteria (Fig. 2), with 102 patients (16.8%) fulfilling five or more criteria simultaneously. In contrast, 75 patients met only one exclusion criterion. Among these, any pathological neuroimaging was the most common single exclusion criterion observed in around 30 patients (around 5% of the total population). Genetic considerations had limited influence on eligibility, as *APOE ε4* homozygosity rarely constituted the sole limiting factor, applying to only 10–11 participants (1.6–1.8% of the total population). The distribution of exclusion domains among individuals with only one exclusion criterion is detailed in Table S5.

At the same time, MRI abnormalities were common overall, affecting nearly 200 (32.3%) participants, most often in combination with other exclusion criteria. Severe white matter disease (Fazekas score 3) was the most frequent imaging-based exclusion (*n* = 156; 25.7%). The substantial overlap among exclusion criteria attenuated the impact of recommendations targeting individual factors.

### Results from sensitivity analyses

Sensitivity analyses demonstrated only modest changes in eligibility estimates across alternative assumptions (Table S4). In the first analysis, removal of age restrictions increased eligibility by one participant for lecanemab. In the most permissive lecanemab scenario, in which both age and BMI restrictions were removed, eligibility increased by three participants (+ 0.3% points). For donanemab, removal of age restrictions resulted in ten additional eligible participants, which increased eligibility with 1.9% points (from 12.8% to 14.7%). In separate analyses applying more inclusive operational definitions of autoimmune disease and malignancy, fewer participants were initially excluded. However, after application of all remaining eligibility criteria in the flowchart, the final number of eligible participants was 88–89 for lecanemab (vs. 86 in the primary analysis) and 79–80 for donanemab (vs. 78), indicating high consistency with the primary analysis and only minor changes in overall eligibility. FDA guidance, but not the AUR, allows anticoagulant use during donanemab therapy. Incorporating this criterion as in sensitivity analysis 6, would make seven additional patients eligible for ATT, increasing the eligibility rate by around 1% point (from 12.8% to 14.0%).

Finally, to assess the impact of high-sensitivity susceptibility imaging, exclusion based on cerebral microbleeds was removed, resulting in only one additional eligible participant.

## Discussion

We found that approximately 13–14% of patients presenting to primary care with cognitive symptoms fulfilled AUR-based clinical eligibility criteria for ATT. Eligible patients were slightly older and included a higher proportion of women than participants in phase 3 ATT trials [30, 31]. Sensitivity analyses demonstrated that eligibility estimates were robust to alternative assumptions regarding physician judgment and more permissive criteria for medication use, with only minimal variation between conservative and permissive scenarios, indicating that real-world eligibility is relatively stable.

Although national regulatory criteria, including stricter MRI-based thresholds [32], may theoretically reduce eligibility in some settings, our sensitivity analyses suggest that such variations are unlikely to materially change overall eligibility estimates. Also, after applying all exclusion criteria, the *APOE ε4/ε4* genotype was relevant for only a small fraction of the cohort (< 2% of the initial population), and excluding *APOE ε4* homozygotes would have reduced eligibility by approximately 1% point.

Our estimates are broadly consistent with previous secondary care studies showing that only a minority of patients with cognitive complaints are eligible for ATT [33–35]. The high prevalence of severe white matter disease in our cohort reflects the real-world setting, where vascular and mixed pathologies are common among older individuals presenting with cognitive symptoms [36].

Eligibility estimates in the present study were lower than those reported in US cohorts (approximately 23%) [37] and in studies focusing on patients with milder cognitive impairment, such as MCI only [33]. These differences likely reflect variation in age, comorbidity burden, healthcare systems, and referral thresholds. Importantly, treatment at earlier disease stages may be associated with both lower risk of symptomatic amyloid-related imaging abnormalities [38] and greater clinical benefit, underscoring the importance of timely identification of eligible patients [39].

An important clinical implication of our findings is that most ineligible patients fulfilled multiple exclusion criteria, many of which, such as relevant comorbidities and anticoagulation therapy, could be identified early in the primary care setting. In our cohort, the initial screening step alone excluded more than 200 of approximately 600 patients. Clearer guidance for primary care physicians regarding early ATT eligibility considerations may help streamline referrals, reduce unnecessary specialist assessments, and manage patient expectations. Future implementation of blood-based biomarkers and optimized brief cognitive screening tools may further refine early triage in primary care.

This study was conducted in a real-world primary care setting and included comprehensive specialist assessments with CSF/PET biomarkers and neuroradiological MRI review, enhancing diagnostic accuracy. Medical records were systematically reviewed by a licensed physician, strengthening the validity of exclusion criteria related to comorbidity and medication use. The robustness of eligibility estimates across sensitivity analyses further strengthens the findings.

Upstream screening and refusal rates were not systematically recorded, which may introduce selection bias. However, inclusions were consecutive and inclusion criteria were broad, supporting the clinical representativeness of the cohort. A substantial proportion of participants had only subjective cognitive complaints. In routine primary care, such mild symptoms rarely prompt further investigation; however, participation in the research study likely led to earlier cognitive assessment and inclusion of individuals at very early stages of disease. This may be considered a strength, as it facilitated early identification, but it may also influence prevalence estimates and diagnostic yield. Consistent with this, the high proportion of patients identified at the MCI stage likely reflects increased awareness of cognitive symptoms and earlier recognition within the BioFINDER–Primary Care framework.

The study was conducted in a single region, potentially limiting generalizability to healthcare systems with different access structures. High-sensitivity 3T MRI may have led to slightly lower eligibility estimates compared with routine clinical settings using 1.5T MRI, although sensitivity analyses regarding microbleeds indicated minimal impact (only one additional eligible participant when not applying the microbleeds criterion). Inter-rater reliability for MRI assessments was not evaluated, which should be considered a limitation, although intra-rater reliability was very high (κ = 0.85–1.00).

Eligibility estimates should be interpreted in light of the parent cohort’s inclusion and exclusion criteria, which may both under- and overestimate eligibility through opposing selection effects. Importantly, clinical eligibility does not equate to treatment initiation. Uptake will depend on shared decision-making, system capacity, and patient preferences. The AUR requirement for a care partner could not be directly assessed, and the lack of systematic data on caregiver availability may have led to an overestimation of real-world eligibility. However, participation in repeated study visits suggests that many participants had access to some level of practical support, such as a caregiver or other assistance if needed.

Several AUR criteria are intentionally broad, which complicates operationalization in observational data. However, our transparent definitions and sensitivity analyses indicate that the main findings are robust. The robustness of eligibility estimates across conservative and permissive scenarios suggest that, even under broader assumptions, the potential treatment population remains small and predictable, supporting more reliable healthcare planning and cost estimations.

## Conclusion

In patients seeking help in primary care due to cognitive symptoms, around 13–14% fulfilled AUR criteria for clinical eligibility for ATT. In routine primary care, patients with comorbidities, medications, or contraindications to MRI that clearly preclude further diagnostic work-up can often be identified. However, key determinations such as objective cognitive staging, final biomarker status, and MRI-based contraindications still require specialist assessment, although initial triaging might be possible in primary care, for example using blood-based biomarkers and cognitive screening. Accordingly, the role of primary care is best conceptualized as recognizing candidates for further diagnostic clarification and prioritizing referral, allowing more efficient use of healthcare resources.

## Supplementary Information

Supplementary Material 1.

Supplementary Material 2. Table S1–S7.

Supplementary Material 3. Figure S1.

## Data Availability

Anonymized data can be shared by request from qualified academic investigators for the purpose of replicating procedures and results presented in the article. Data transfer is required to be in agreement with EU legislation on the general data protection regulation and decisions by the Ethical Review Board of Sweden and Region Skåne, which should be regulated in a material transfer agreement.

## Abbreviations

ABRA: Aβ-related angiitis
AD: Alzheimer’s disease
ADL: Activity of Daily Living
ARIA: Amyloid Related Imaging Abnormalities
ATC: Anatomical Therapeutic Chemical classification system
ATT: Amyloid-targeting therapies
AUR: Appropriate Use Recommendations
BMI: Body Mass Index
CAA-ri: Cerebral Amyloid Angiopathy-related inflammation
CSF: Cerebrospinal fluid
EMA: The European Medicines Agency
FDA: U.S. Food and Drug Administration
ICD-10: International Classification of Disease
MCI: Mild Cognitive Impairment
MMSE: Mini-Mental State Examination
MRI: Magnetic Resonance Imaging
SCD: Subjective Cognitive Decline
STROBE: Strengthening the Reporting of Observational Studies in Epidemiology
TIA: Transient Ischemic Attack
PET: Positron Emission Tomography
WMH: White Matter Hyperintensities
