# Supplementary material for: Eligibility for amyloid targeting therapies among primary care patients with cognitive symptoms
Source: Alzheimers Res Ther. 2026 Mar 21;18:77. doi: 10.1186/s13195-026-02019-2 (PMC13064071; doi:10.1186/s13195-026-02019-2)
Supplement: Supplementary file 1 — Supplementary Material 1. [file 13195_2026_2019_MOESM1_ESM.docx]

**SUPPLEMENTARY MATERIAL**

*S1. Swedish primary care pathway for patients with cognitive symptoms*

In Sweden, primary care serves as the main entry point to the healthcare system for individuals with cognitive complaints. Patients experiencing memory problems or other cognitive symptoms typically seek initial evaluation through their general practitioner (GP), who performs a first-line clinical assessment and initiates a standardized basic dementia work-up. Specialist psychiatric services are primarily involved when affective, functional, or other primary psychiatric conditions are suspected, rather than in the routine evaluation of suspected neurodegenerative disease. Referral to secondary or tertiary specialist memory clinics is generally reserved for patients with early-onset symptoms, atypical clinical features or diagnostic uncertainty. Within this healthcare context, patients undergoing cognitive evaluation in primary care represent a broad spectrum of individuals presenting with cognitive symptoms.

*S2. Cognitive staging*Subjective Cognitive Decline (SCD) was defined as experiencing cognitive symptoms to the level that led the patient to seek help in primary care but not fulfilling the criteria for Mild Cognitive Impairment (MCI) or dementia. MCI was diagnosed based on the presence of notable cognitive symptoms and abnormal cognitive test results using the RBANS (Repeatable Battery for the Assessment of Neuropsychological Status) battery, accounting for premorbid cognitive level. The MCI definition did not require that a strict threshold in a cognitive domain was met (although all performed <−1 SD in at least one cognitive domain in the RBANS battery) but was based on the overall clinical assessment. The classification followed the design of the MCI classification of the Mayo Clinic Study of Aging (1) and was in line with the DSM-5 criteria for mild neurocognitive disorder (2). Dementia was diagnosed according to the DSM-5 criteria for major neurocognitive disorder. The differentiation between mild and moderate dementia followed the Alzheimer’s Association staging for clinical stage 4 (“mild dementia”) and clinical stage 5 (“moderate dementia”), where stage 5 states dependence for basic activities of daily living (3).

*S3. Definition of AD etiology*Alzheimer’s Disease (AD) was defined as having a clinical syndrome of AD (according to the IWG criteria) at the MCI or dementia stage and AD biomarker confirmation, either a positive Cerebrospinal fluid (CSF) Aβ42/40 result or an amyloid PET examination (visual read). Patients with positive AD biomarkers but symptoms suggestive of another etiology, such as frontotemporal dementia, Lewy body disease, etc., were not coded as AD. In patients with biomarker verification of multiple etiologies, for example AD in CSF and significant vascular pathology on MRI, the decision was based on clinical judgment regarding the driving pathology of the symptoms. In unclear cases, the group suggested appropriate additional examinations. These could, for example, include FDG-PET, DAT-PET, tau-PET, a lumbar tap test, or additional neuropsychological assessment.
CSF p-tau217 was measured in participants who underwent lumbar puncture and used as a measure of tau status (T1) according to the 2024 Alzheimer’s Association criteria for AD. A previously published cutoff was used to define T1 positivity (>11.42 pg/mL) (4).

**REFERENCES**

1. Roberts RO, Geda YE, Knopman DS, Cha RH, Pankratz VS, Boeve BF, et al. The Mayo Clinic Study of Aging: design and sampling, participation, baseline measures and sample characteristics. Neuroepidemiology. 2008;30(1):58–69.

2. 5th ed. Arlington: American Psychiatric Association; 2013. American Psychiatric Association: Diagnostic and Statistical Manual of Mental Disorders.

3. Jack CR, Jr., Andrews JS, Beach TG, Buracchio T, Dunn B, Graf A, et al.

Revised criteria for diagnosis and staging of Alzheimer's disease: Alzheimer's Association Workgroup. Alzheimers Dement. 2024;20(8):5143–694.

4. Palmqvist S, Tideman P, Mattsson-Carlgren N, Schindler SE, Smith R, Ossenkoppele R, et al. Blood Biomarkers to Detect Alzheimer Disease in Primary Care and Secondary Care. JAMA. 2024;332(15):1245–57.
