# Supplementary material for: Eligibility for amyloid targeting therapies among primary care patients with cognitive symptoms
Source: Alzheimers Res Ther. 2026 Mar 21;18:77. doi: 10.1186/s13195-026-02019-2 (PMC13064071; doi:10.1186/s13195-026-02019-2)
Supplement: Supplementary file 2 — Supplementary Material 2. Table S1–S7. [file 13195_2026_2019_MOESM2_ESM.docx]

**Supplement Tables**

| **Table S1. STROBE Statement—checklist of items that should be included in reports of observational studies** | | | | |
| --- | --- | --- | --- | --- |
|  | Item No | Recommendation |  |  |
| **Title and abstract** | 1 | (*a*) Indicate the study’s design with a commonly used term in the title or the abstract |  | Yes page 2 |
|  |  | (*b*) Provide in the abstract an informative and balanced summary of what was done and what was found |  | Yes page 2 |
| Introduction | | |  |  |
| Background/rationale | 2 | Explain the scientific background and rationale for the investigation being reported |  | Yes page 3-4 |
| Objectives | 3 | State specific objectives, including any prespecified hypotheses |  | Yes page 5 |
| Methods | | |  |  |
| Study design | 4 | Present key elements of study design early in the paper |  | Yes page 5 |
| Setting | 5 | Describe the setting, locations, and relevant dates, including periods of recruitment, exposure, follow-up, and data collection |  | Yes page 5-6 |
| Participants | 6 | (*a*) *Cohort study*—Give the eligibility criteria, and the sources and methods of selection of participants. Describe methods of follow-up |  | Yes page 5-6 |
|  |  | (*b*) *Cohort study*—For matched studies, give matching criteria and number of exposed and unexposed |  | N/A |
| Variables | 7 | Clearly define all outcomes, exposures, predictors, potential confounders, and effect modifiers. Give diagnostic criteria, if applicable |  | Yes page 8-9 |
| Data sources/ measurement | 8* | For each variable of interest, give sources of data and details of methods of assessment (measurement). Describe comparability of assessment methods if there is more than one group |  | Yes page 8-9 |
| Bias | 9 | Describe any efforts to address potential sources of bias |  | Yes page 10 |
| Study size | 10 | Explain how the study size was arrived at |  | Yes page 11 |
| Quantitative variables | 11 | Explain how quantitative variables were handled in the analyses. If applicable, describe which groupings were chosen and why |  | Yes page 10 |
| Statistical methods | 12 | (*a*) Describe all statistical methods, including those used to control for confounding |  | Yes page 10 |
|  |  | (*b*) Describe any methods used to examine subgroups and interactions |  | Yes page 10 |
|  |  | (*c*) Explain how missing data were addressed |  | Yes page 5 |
|  |  | (*d*) *Cohort study*—If applicable, explain how loss to follow-up was addressed |  |  |
|  |  | (*e*) Describe any sensitivity analyses |  | Yes page 10 |

| Participants | 13* | (a) Report numbers of individuals at each stage of study—eg numbers potentially eligible, examined for eligibility, confirmed eligible, included in the study, completing follow-up, and analysed | Yes, Fig 1 and SFig1 |
| --- | --- | --- | --- |
|  |  | (b) Give reasons for non-participation at each stage |  |
|  |  | (c) Consider use of a flow diagram | Yes Fig 1 and SFig1 |
| Descriptive data | 14* | (a) Give characteristics of study participants (eg demographic, clinical, social) and information on exposures and potential confounders | Yes p11, Table 2 |
|  |  | (b) Indicate number of participants with missing data for each variable of interest | N/A |
|  |  | (c) *Cohort study*—Summarise follow-up time (eg, average and total amount) | N/A |
| Outcome data | 15* | *Cohort study*—Report numbers of outcome events or summary measures over time | N/A |
| Main results | 16 | (*a*) Give unadjusted estimates and, if applicable, confounder-adjusted estimates and their precision (eg, 95% confidence interval). Make clear which confounders were adjusted for and why they were included | N/A |
|  |  | (*b*) Report category boundaries when continuous variables were categorized | Yes table2 and table 3 |
|  |  | (*c*) If relevant, consider translating estimates of relative risk into absolute risk for a meaningful time period | N/A |
| Other analyses | 17 | Report other analyses done—eg analyses of subgroups and interactions, and sensitivity analyses | Yes page 11 |
| Discussion | | |  |
| Key results | 18 | Summarise key results with reference to study objectives | Yes page 11 |
| Limitations | 19 | Discuss limitations of the study, taking into account sources of potential bias or imprecision. Discuss both direction and magnitude of any potential bias | Yes page 16 |
| Interpretation | 20 | Give a cautious overall interpretation of results considering objectives, limitations, multiplicity of analyses, results from similar studies, and other relevant evidence | Yes page 15 |
| Generalisability | 21 | Discuss the generalisability (external validity) of the study results | Yes page 16 |
| Other information | | |  |
| Funding | 22 | Give the source of funding and the role of the funders for the present study and, if applicable, for the original study on which the present article is based | Yes page 20 |
| *Give information separately for exposed and unexposed groups  **Note:** An Explanation and Elaboration article discusses each checklist item and gives methodological background and published examples of transparent reporting. The STROBE checklist is best used in conjunction with this article (freely available on the Web sites of PLoS Medicine at http://www.plosmedicine.org/, Annals of Internal Medicine at http://www.annals.org/, and Epidemiology at http://www.epidem.com/). Information on the STROBE Initiative is available at www.strobe-statement.org. | | | |

| **Table S2. Definitions and alternative operations of exclusion criteria** | | |
| --- | --- | --- |
| **Exclusion criteria** | **The AUR interpretation in primary analysis** | **More permissive physician-judgment scenarios in sensitivity analyses** |
|  |  |  |
| **Seizures:**  ATC code:  *Antiepileptic drugs N03A* | The use of active anti-epileptic medication. (The AUR states: any history of seizures; however, this was not directly applicable to the study data) | Ongoing treatment with antiepileptic medication. |
| **Autoimmune disease:** ICD codes: *Connective tissue disease M32–M35. Inflammatory arthritis M05–M07, M45–M46*  *IBD K50–K51. Neuroinflammatory disease G35. Vasculitis M30–M31*  ATC-codes: *Immunoglobulins J06BA01–J06BA02*  *Monoclonal antibodies & targeted immunomodulators L04AA, L04AB, L04AC*  *Conventional systemic immunosuppressants L04AX, L04AD, L01AA*  *Plasmapheresis (procedure), KVÅ: DR028* | Any autoimmune disease or ongoing immunosuppressive treatment | Any immunological disease which is not adequately controlled, or which requires treatment with immunoglobulins, systemic monoclonal antibodies (or derivatives of monoclonal antibodies), systemic immunosuppressants, or plasmapheresis. |
| **Cancer**  ICD codes:  *Malignant neoplasms C00–C80, C97*  *Hematologic malignancies C81–C96*  *(Non-melanoma skin cancers C44, carcinoma in situ D00–D09 excluded)* | Active cancer that interferes with the ability to comply with treatment. Active cancer defined as history of cancer 5 years, except for non-metastatic basal and/or squamous cell carcinoma of the skin, nonprogressive prostate cancer, or other cancers with low risk of recurrence or spread. | Patients with cancer were clinically assessed as having stable disease and were therefore eligible for inclusion in the BioFINDER cohort despite having a registered cancer diagnosis. |
| **Anticoagulant use**  ATC code:  B01A | Patients on anticoagulants (tPA should not be administered to individuals receiving lecanemab) | Anticoagulant treatment is permitted. |
|  |  |  |

| **Table S3. Baseline demographics of the excluded population** | |
| --- | --- |
| **Excluded population (n, %)** | 238 (100%) |
| Age, years, n=162 (mean ± SD) | 77.1 ± 7.6 |
| Sex, female n=155 (%) | 84 (54.2%) |
| BMI, n=79 (mean ± SD) | 26.7 ± 5.1 |
| Education level, years, n= 131 (mean ± SD) | 10.8 ± 3.0 |
| MMSE, n=199 (mean ± SD) | 25.6 ± 3.6 |
| MoCA, n=166 (mean ± SD) | 20.7 ± 4.2 |
| n indicates the number of participants with available data for each variable | |

| **Table S4. Sensitivity analyses of different scenarios** | | |
| --- | --- | --- |
| **Scenario** | **Eligible patients for lecanemab n (%)** | **Eligible patients for donanemab n (%)** |
| Primary analysis | 86 (14.2%) | 78 (12.8%) |
| Sensitivity analysis 1 (No age limit) | 87 (14.3%) | 89 (14.7%) |
| Sensitivity analysis 2 (No BMI limit) | 88 (14.5%) | N/A |
| Sensitivity analysis 3 (No age or BMI limit) | 89 (14.7%) | 89 (14.7%) |
| Sensitivity analysis 4 (Active autoimmune disease requiring systemic immunosuppressive treatment, instead of any autoimmune disease) | 88 (14.5%) | 79 (13.0%) |
| Sensitivity analysis 5 (Cancer patients not excluded) | 87 (14.3%) | 79 (13.0%) |
| Sensitivity analysis 6 (Patients on anticoagulant treatment not excluded) | 93 (15.3%) | 85 (14.0%) |
| Sensitivity analysis 7 (Microbleeds not excluded) | 87 (14.3%) | 79 (13.0%) |

| **Table S5. Individuals excluded by a single criterion** | | |
| --- | --- | --- |
| **Exclusion criterion** | **Count of total population, n (%)**  **(lecanemab AUR)** | **Count of total population, n (%) (donanemab AUR)** |
| Any pathological neuroimaging, n (%) | 32 (5.3%) | 28 (4.6%) |
| Any comorbidity, n (%) | 14 (2.3%) | 11 (1.8%) |
| Non-AD etiology, n (%) | 12 (2.0%) | 10 (1.6%) |
| *APOE* ε4 homozygous, n (%) | 11 (1.8%) | 10 (1.6%) |
| Ineligible cognitive status, n (%) | 4 (0.7%) | 3 (0.5%) |
| Age and/or BMI, n (%) | 2 (0.3%) | 10 (1.6%) |
| Total individuals with one exclusion criterion, n (%) | 75 (12.4%) | 72 (11.9%) |

| **Table S6. Weighted Cohen’s kappa for intra-rater reliability of MRI visual assessments (n = 83)** | |
| --- | --- |
| **Assessment** | **Weighted Kappa Score** |
| Fazekas scores | 0.993 |
| Fazekas 3 | 1.000 |
| >2 Lacunes | 1.000 |
| >4 Microbleeds | 0.851 |
| Macrohemorrhage >10mm | 0.851 |
| Cortical siderosis | 0.851 |
| Vascular edema | 1.000 |
| Other major neuropathology | 1.000 |
| Final eligibility | 1.000 |
| Statistical significance of weighted kappa values was assessed separately; all values were statistically significant (p < 0.05). | |

| **Table S7. CSF p-tau217 levels and status stratified by CSF amyloid status and age (n=523)** | | | |
| --- | --- | --- | --- |
|  | **All** | **Amyloid positive, n=283** | **Amyloid negative, n=240** |
| CSF p-tau217, pg/mL, mean (SD) | 23.20 (25.55) | 36.83 (28.08) | 7.13 (4.05) |
| Age <65, mean (SD) | 10.81 (13.05) | 25.03 (16.39) | 4.89 (3.65) |
| Age 65-80 mean (SD) | 23.54 (28.15) | 38.69 (32.36) | 7.40 (4.26) |
| Age >80 mean (SD) | 26.64 (21.51) | 35.56 (20.78) | 7.89 (3.04) |
|  |  |  |  |
| CSF p-tau217 positivity (T_1_+) (n, %) | 286 (54.68%) | 255 (90.11%) | 31 (12.92%) |
| Age <65, n (%) | 13 (25.49%) | 10 (66.67%) | 3 (8.33%) |
| Age 65-80 , n (%) | 173 (54.06%) | 151 (91.52%) | 22 (14.19%) |
| Age >80 , n (%) | 100 (65.79%) | 94 (91.26%) | 6 (12.24%) |

p-tau levels and t-tau status (positivity defined as >11.42 pg/mL) for all individuals with available CSF data (n = 523).
