# Supplementary figures and images for: Eligibility for amyloid targeting therapies among primary care patients with cognitive symptoms
Source: Alzheimers Res Ther. 2026 Mar 21;18:77. doi: 10.1186/s13195-026-02019-2 (PMC13064071; doi:10.1186/s13195-026-02019-2)

**Supplement Figure**


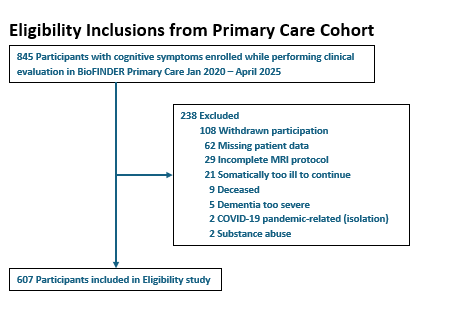


**Figure S1.** Flowchart of inclusion and study enrollment

Supplement: Supplementary file 3 — Supplementary Material 3. Figure S1. [file 13195_2026_2019_MOESM3_ESM.docx]
